# Risk Factors Associated With Pediatric Asthma Revisits to Emergency Departments

**DOI:** 10.1016/j.focus.2026.100520

**Published:** 2026-06-15

**Authors:** Nima Khodakarami, Gogoal Falia, Hye Jin Park, Marvellous Akinlotan, Alva O. Ferdinand

**Affiliations:** 1 Penn State University, Monaca, Pennsylvania; 2 Center for Applied Studies in Health Economics, Penn State College of Medicine, Hershey, Pennsylvania; 3 Texas A&M University School of Public Health, College Station, Texas; 4 Southwest Rural Health Research Center, College Station, Texas; 5 University of Utah School of Dentistry, Salt Lake City, Utah; 6 Texas A&M University College of Dentistry, Dallas, Texas

**Keywords:** Pediatric asthma, emergency departments, readmissions, race and ethnicity, Black and Hispanic children, rural

## Abstract

**Introduction:**

Asthma is one of the most common reasons for emergency department visits among children in the U.S. Although prior studies have found sociodemographic disparities in asthma revisits, most focus on short follow-up periods or single-state data, limiting the understanding of persistent emergency department reliance over time. The purpose of this study was to fill this gap and understand the associations between sociodemographic characteristics and the likelihood of emergency department revisits.

**Methods:**

This observational retrospective cohort study used emergency department data from the states of Florida, Maryland, and Wisconsin for the years 2016–2019. The study identified a sample of children aged 0–17 years with asthma visits in 2016 and observed their asthma emergency department usage over time. Logistic regression was used to identify predictors of emergency department revisits for the 2016 asthma cohort.

**Results:**

Among 38,331 children with an asthma-related emergency department visit in 2016, 32% of the 2016 cohort returned to the emergency department for additional asthma visits between 2017 and 2019, with most revisits occurring in 2017. In adjusted analyses, Black and Hispanic children as well as younger children, particularly those aged 5–11 years, had higher odds of revisits for asthma. Other factors that contributed to higher odds of asthma emergency department revisits included being male, having Medicaid insurance, living in lower-income neighborhoods, and living in large metropolitan areas.

**Conclusions:**

Persistent sociodemographic and geographic disparities exist in asthma emergency department readmissions.

## Highlights

• Longitudinal data track pediatric asthma emergency visits in 3 U.S. states.
• Black and Hispanic children face higher odds of repeated visits.
• Medicaid status and urban residence are key predictors of repeat visits.
• Most repeat visits occur within 1 year of the initial visit.
• Children aged 5–11 years carry the highest burden of repeat visits.

## INTRODUCTION

Children with asthma recurrently use the emergency department (ED) for medical care. Every year, about half a million children visit the ED for asthma, analogous to 75 visits per 10,000 children.^1^ For some children, these visits are not single events. Rather, revisits to the ED among children with asthma are common.^2^^,^^3^ Therefore, reducing the burden of such revisits on health care and the impact it can have on parental employment and the child’s ability to succeed in school remains an objective of the nation’s health.^4^^,^^5^^,^^6^^,^^7^ Current studies show that factors predictive of asthma ED revisits include sociodemographic risk factors such as patients’ race/ethnicity, types of insurance, and patient location. In multiple studies, the authors found that Black and Hispanic children are more likely to revisit the ED than White children. These studies further found that children covered by Medicaid are more likely to revisit the ED than those with private insurance.^8^^,^^9^^,^^10^ Moreover, the research showed that children in urban areas are more likely to revisit the ED for the treatment of asthma than those in nonurban locations.^11^^,^^12^

These findings, which are based on analyses of short-term return (7–30 days) to the ED after the initial visit, mainly aim to underscore the importance of outpatient follow-up care after initial visits in reducing the risk of repeat ED use for asthma among the identified patients.^3^^,^^8^^,^^13^, ^14^, ^15^, ^16^, ^17^, ^18^ However, outpatient follow-up care does not appear to be associated with a decreased likelihood of asthma-related ED revisits in 1 year or more.^3^^,^^19^ Instead, other factors, such as patient sociodemographic characteristics; access barriers; environmental conditions; and other social, cultural, and policy factors, may contribute to the persistence of ED reliance.^20^^,^^21^ Nevertheless, there is no study that examines the characteristics of patients who persistently rely on the ED for asthma management.

This study investigates the risk factors associated with revisits to the ED among children who had asthma from 2016 through 2019. The study focuses on children aged <18 years with visits for asthma and examines data from states that aim to reduce pediatric asthma rates, including Florida (FL), Maryland (MD), and Wisconsin (WI). The selection of states is based on the availability of data that allowed tracking of children across the years of the study period and observation of their ED use for asthma.

The findings of this study will be of interest to those involved in the management of asthma, particularly for FL, MD, and WI, and may be instructive for policymakers and healthcare systems. By identifying patients who persistently go to the ED for asthma care, these parties can more effectively target interventions that address the unmet health and social needs of children most at risk of revisits and ultimately reduce ED utilization.^22^^,^^23^ Reducing persistent reliance on the ED is desirable for optimizing scarce healthcare resources, particularly where the demand for ED services exceeds available capacity and the ED struggles to meet benchmarks for emergency treatment performance.^24^

## METHODS

### Study Sample

This retrospective analysis used data from the State Emergency Department Databases (SEDD) for 2016 through 2019, available through the Healthcare Cost and Utilization Project (HCUP). The data set provides information on ED encounters with a set of clinical (primary and secondary diagnosis codes) and nonclinical information on all patients, including race/ethnicity (e.g., White, Black, Hispanic, Asian or Pacific Islander, Native American, and other), sex (e.g., male and female), age in years (at the time of admission), insurance status (e.g., Medicare, Medicaid, private insurance, uninsured, self-pay, no charge, and other), median income of the patient's ZIP code assigned to 4 quartiles (e.g., 1 indicates the poorest, whereas 4 indicates the wealthiest populations), and patient location captured by a 6-category urban–rural classification subdivided by population (e.g., 1=central metropolitan [≥1 million population], 2=fringe metropolitan [≥1 million population], 3=medium metropolitan [250–999 thousand population], 4=small metropolitan [50–249 thousand population], 5=micropolitan, 6=nonmetropolitan/nonmicropolitan or noncore). Data from FL, MD, and WI include a unique record linkage number (VisitLink) for each patient that allowed tracking of children’s ED usage across time.^25^ Population demographic data (e.g., age, sex, and race/ethnicity) at the county level were also collected from the American Community Survey for the years 2016 through 2019.^26^

A sample of children aged 0–17 years with asthma as the primary diagnosis was identified, defined by the ICD-10-CM code of J45.^27^ Patients without a linkage number were excluded, because they could not be followed over the years. Children residing outside the state and those with missing sex, age, quarter, principal diagnosis, and county were also excluded from the study sample.

### Measures

The primary outcomes were binary variables indicating whether the 2016 cohort returned to the ED for asthma visits during the years 2017–2019. To construct these outcomes, first, children aged 0–17 years who had an ED visit for asthma in 2016 were identified; this year was treated as the index year, and all other patients were excluded. Three binary outcomes were then created, each defining whether a patient in the 2016 cohort returned to the ED in any of the years from 2017 to 2019 for asthma visits. Those who visited the ED only in 2016, with no subsequent visits in the following years, were the reference group and were assigned a value of 0. Children who visited the ED in 2016 and revisited the ED for asthma-related care within 1, 2, or 3 years between 2017 and 2019 were assigned a value of 1. Specifically, the 1‑year outcome was coded as 1 if the child had at least 1 asthma‑related ED revisit within any single calendar year during follow‑up (i.e., 2017, 2018, or 2019) and 0 otherwise. Similarly, the 2‑year outcome was coded as 1 if the child revisited the ED for asthma during any 2‑year period (2017–2018, 2018–2019, or 2017–2019) and 0 otherwise. The 3‑year outcome indicated whether the child had at least 1 asthma‑related ED revisit during the full 3‑year follow‑up period from 2017 through 2019.

### Statistical Analysis

Descriptive analysis was employed to identify children with occasional ED use for asthma. Logistic regression was used to identify predictors of ED revisits for the 2016 asthma cohort. Three regressions were conducted, each comparing those with an asthma ED revisit with those who visited the ED for asthma only in 2016. Therefore, in each regression, the reference group consisted of patients with ED use in 2016 who had no ED visits in successive years. Covariates were observed in 2016 and included patients’ race/ethnicity (White, Black, Hispanic, and other race, where Asian or Pacific Islander, Native American, and other were grouped as other race), sex (male and female), age (grouped as 0–4, 5–11, 12–14, and 15–17 years), insurance status (private, Medicaid, uninsured, and other public coverages, where self-pay and no charge were grouped as uninsured, and a few Medicare cases were grouped with other public coverages), median household income in the patient’s ZIP code (quartiles identified by a value of 1–4, indicating the poorest to the wealthiest populations), and residence in an urban or rural county (using a 6-category urban–rural classification scheme for U.S. counties).^28^ A variable indicating how many times a patient changed coverage over the years was created and included in the model. The Elixhauser Comorbidity Index was also used as a covariate to account for differences in underlying health status.^29^ County-level demographic characteristics and state-fixed effects were also included in the model. Each model was estimated at the child level, with year-quarter fixed effects pooled across states to control for common temporal trends. SEs were clustered at the county level to account for within-county correlation. Results were presented as ORs with 95% CIs. A *p*<0.05 was set to denote statistical significance. STATA, Version 18.0, was used for all analyses. This study was deemed exempt from review by the IRB at Texas A&M University.

## RESULTS

A total of 38,331 children were identified in 2016 who visited the ED for asthma. As Table 1 shows, children who were Black, male, aged 5–11 years, covered by Medicaid, residing in ZIP codes with the lowest median household income quartile, and living in large metropolitan areas accounted for most of the 2016 cohort. The majority of patients who returned to the ED in the following years were also from these groups of children.

**Table 1:** Characteristics of Children With Occasional ED Visits and Revisits Between 2016 and 2019

Characteristics	All children	Index visits: ED visits in 2016 with no return	Return to ED for 1 year	Return to ED for 2 years	Return to ED for 3 years
Total visits	38,331	25,988	8,078	3,064	1,201
Race/ethnicity (%)					
White	9,260 (24)	7,011 (27)	1,615 (20)	468 (15)	166 (14)
Black	19,191 (50)	11,953 (46)	4,527 (56)	1,909 (62)	802 (67)
Hispanic	7,993 (21)	5,617 (22)	1,590 (20)	583 (19)	203 (17)
Other	1,617 (4)	1,205 (5)	296 (4)	92 (3)	24 (2)
Missing	270 (<1)	202 (<1)	50 (<1)	12 (<1)	6 (<1)
Sex (%)					
Male	22,938 (60)	15,481 (60)	4,883 (60)	1,866 (60)	708 (60)
Female	15,393 (40)	10,507 (40)	3,195 (40)	1,198 (40)	493 (40)
Age group (%)					
Age 0–4	11,433 (30)	7,837 (30)	2,418 (30)	833 (27)	346 (29)
Age 5–11	18,198 (47)	12,069 (47)	3,896 (48)	1,620 (53)	612 (51)
Age 12–14	4,487 (12)	3,165 (12)	912 (11)	307 (10)	103 (9)
Age 15–17	4,213 (11)	2,917 (11)	852 (11)	304 (10)	140 (12)
Type of insurance (%)					
Private	6,762 (18)	5,236 (20)	1,100 (14)	318 (10)	108 (9)
Medicaid	29,426 (77)	19,124 (74)	6,625 (82)	2,629 (86)	1,048 (87)
Uninsured	1,119 (3)	865 (3)	176 (2)	58 (2)	20 (2)
Other	1,020 (3)	761 (3)	176 (2)	58 (2)	25 (2)
Median income (%)					
Quartile 1	17,173 (45)	11,029 (43)	3,947 (49)	1,549 (51)	648 (54)
Quartile 2	9,480 (25)	6,493 (25)	1,944 (24)	757 (25)	286 (24)
Quartile 3	7,208 (19)	5,125 (20)	1,399 (18)	498 (16)	186 (16)
Quartile 4	4,215 (11)	3,159 (12)	743 (9)	244 (8)	69 (6)
Urban/rural (%)					
Central large metropolitan	13,881 (36)	8,788 (34)	3,235 (40)	1,317 (43)	541 (45)
Fringe large metropolitan	12,774 (33)	9,185 (35)	2,459 (30)	807 (27)	323 (27)
Medium metropolitan	7,454 (19)	4,979 (19)	1,581 (20)	636 (21)	258 (21)
Small metropolitan	2,195 (6)	1,540 (6)	443 (6)	169 (5)	43 (4)
Micropolitan	1,048 (3)	761 (3)	192 (2)	75 (2)	20 (2)
Noncore	979 (2)	737 (3)	167 (2)	59 (2)	16 (1)

ED, emergency department.

The pattern of revisits is shown in Table 2. Of the total cohort of children receiving asthma-related care in the ED in 2016 (*n*=38,331), 68% (*n*=25,988) visited the ED only in 2016 and did not have any subsequent visits in the following years. About 32% of the 2016 cohort (*n*=12,343) returned to the ED for additional asthma visits at least once between 2017 and 2019. Notably, 21% (*n*=8,078) had 1 ED revisit in 1 of the 3 subsequent years, 8% (*n*=3,064) had ED revisits in 2 of the 3 subsequent years, and 3% (*n*=1,201) had ED revisits in all 3 subsequent years during the study period. Table 2 depicts that the majority of returns to the ED occurred in 2017 (2017: 7,873 children; 21% of the cohort), 1 year after the initial visits in 2016.

**Table 2:** Pattern of Revisits in Children Going to ED for Asthma Between 2016 and 2019 (N=38,331)

Visit year(s)	Index visits: ED visits in 2016 with no return	Return to ED for 1 year	Return to ED for 2 years	Return to ED for 3 years	Total
2016 only	25,988				25,988
2016 and 2017		4,383			4,383
2016 and 2018		2,141			2,141
2016 and 2019		1,554			1,554
2016, 2017, and 2018			1,419		1,419
2016, 2017, and 2019			870		870
2016, 2018, and 2019			775		775
2016, 2017, 2018, and 2019				1,201	1,201
Total	25,988	8,078	3,064	1,201	38,331

ED, emergency department.

Table 3 shows the results of the logistic regression analyses. In all regressions, the reference group consisted of children who visited the ED for asthma-related care only in 2016. The results of the first regression are presented in the first column, showing the odds of children returning to the ED in 2017, 2018, or 2019 for asthma. The findings show that Black children were associated with higher odds (OR=1.39, 95% CI=1.25, 1.53) of having ED visits than White children. Females were associated with lower odds of returning to the ED than males (OR=0.93, 95% CI=0.87, 0.99). In terms of age, younger children aged 0–4 years (OR=0.15, 95% CI=1.01, 1.30) and 5–11 years (OR=1.26, 95% CI=1.12, 1.41), and 12–14 years (OR=1.68, CI=1.48, 1.91) were associated with higher odds of returning to the ED than those aged 15–17 years. With respect to insurance status, children covered by Medicaid (OR=1.16, 95% CI=1.09, 1.25) were associated with higher odds, and those uninsured were associated with lower odds (OR=0.84; 95% CI=0.75, 0.94) of revisiting the ED than privately insured individuals. With respect to income, patients residing in the lowest-income ZIP code quartile were associated with higher odds (OR=1.10, 95% CI=1.00, 1.20) of revisiting the ED than those living in the highest income quartile (wealthiest). Considering patient location, children from large fringe metropolitan (OR=0.86, 95% CI=0.77, 0.96), medium metropolitan (OR=0.85, 95% CI=0.76, 0.95), micropolitan (OR=0.70, 95% CI=0.53, 0.92), and noncore (OR=0.65, 95% CI=0.49, 0.86) areas were associated with lower odds of revisiting the ED than children residing in large central metropolitan areas.

**Table 3:** Odds of Revisits for Occasional ED Use for the 2016 Cohort in Years 2017 and 2019

	Return to ED for 1 Year	Return to ED for 2 Years	Return to ED for 3 Years
Race/ethnicity			
White	1.00	1.00	1.00
Black	1.39*** (1.25, 1.53)	2.05*** (1.81, 2.32)	2.43*** (1.94, 3.04)
Hispanic	1.12 (0.99, 1.26)	1.41*** (1.23, 1.62)	1.41* (1.07, 1.85)
Other	1.08 (0.91, 1.28)	1.33* (1.06, 1.53)	1.17 (0.85, 1.61)
Sex			
Male	1.00	1.00	1.00
Female	0.93* (0.87, 0.99)	0.87** (0.79, 0.96)	0.92 (0.80, 1.04)
Age group, years			
Age 0–4	1.15* (1.01, 1.30)	1.19* (1.02, 1.38)	1.15 (0.84, 1.56)
Age 5–11	1.26*** (1.12, 1.41)	1.68*** (1.42, 1.99)	1.78*** (1.45, 2.20)
Age 12–14	1.10 (0.99, 1.21)	1.18* (1.02, 1.36)	1.17* (0.85, 1.82)
Age 15–17	1.00	1.00	1.00
Type of insurance			
Private	1.00	1.00	1.00
Medicaid	1.16*** (1.09, 1.25)	1.36*** (1.20, 1.53)	1.58*** (1.33, 1.88)
Uninsured	0.84** (0.75, 0.94)	0.90 (0.78, 1.04)	0.74* (0.57, 0.95)
other	0.88 (0.73, 1.06)	0.82 (0.62, 1.09)	1.24 (0.85, 1.82)
Median income			
Quartile 1	1.10* (1.00, 1.20)	1.09 (0.91, 1.29)	1.51** (1.13, 1.87)
Quartile 2	1.04 (0.94, 1.15)	1.06 (0.90, 1.27)	1.28 (0.93, 1.76)
Quartile 3	0.99 (0.90, 1.09)	0.97 (0.84, 1.13)	1.08 (0.76, 1.53)
Quartile 4	1.00	1.00	1.00
Urban/rural			
Central large metropolitan	1.00	1.00	1.00
Fringe large metropolitan	0.86* (0.77, 0.96)	0.77*** (0.67, 0.88)	0.84 (0.66, 1.07)
Medium metropolitan	0.85*** (0.76, 0.95)	0.88 (0.73, 1.07)	0.99 (0.78, 1.27)
Small metropolitan	0.83 (0.68, 1.02)	0.89 (0.71, 1.12)	0.57* (0.38, 0.87)
Micropolitan	0.70* (0.53, 0.92)	0.70* (0.52, 0.94)	0.45** (0.26, 0.76)
Noncore	0.65** (0.49, 0.86)	0.51*** (0.34, 0.75)	0.34*** (0.18, 0.62)

*Note*: Boldface indicates statistical significance (*p<0.05, **p<0.01, and ***p<0.001).

Results are from multivariable logistic regressions using the HCUP-SEDD data for the years 2016–2019. Each column represents a single regression. Models were adjusted for county characteristics, underlying comorbidities (Elixhauser Comorbidity Index), and changes in insurance. SEs were clustered at the county level. Confidence intervals are in parentheses.

ED, emergency department; HCUP, Healthcare Cost and Utilization Project; SEDD, State Emergency Department Databases.

The second column presents the results of the second regression, indicating the odds of the 2016 cohort returning to the ED for asthma visits in any 2 follow-up years between 2017 and 2019, compared with children who had only a single asthma-related ED visit in 2016. The results indicate that being Black (OR=2.05, 95% CI=1.81, 2.32) and Hispanic (OR=1.41, 95% CI=1.23, 1.62) was associated with higher odds of asthma-related revisits than being White. Being female was associated with lower odds of returning to the ED than being male (OR=0.87, 95% CI=0.79, 0.96). In terms of age, younger children aged 0–4 years (OR=1.19, 95% CI=1.02, 1.38), 5–11 years (OR=1.68, 95% CI=1.42, 1.99), and 12–14 years (OR=1.18, 95% CI=1.02, 1.36) were associated with higher odds of asthma-related revisits than those aged 15–17 years. With respect to insurance coverage, being covered by Medicaid (OR=1.36, 95% CI=1.20, 1.53) was associated with higher odds of revisiting the ED than being privately insured. Moreover, children from large fringe metropolitan areas (OR=00.77, 95% CI=0.67, 0.88), micropolitan areas (OR=0.70, 95% CI=0.52, 0.94), and noncore areas (OR=0.51, 95% CI=0.34, 0.75) were associated with lower odds of revisiting the ED than children residing in large central metropolitan areas.

The results of the third regression are shown in the fourth column. They present the likelihood of the 2016 cohort returning to an ED for asthma in all 3 follow-up years (2017, 2018, and 2019). The results show that children who were Black (OR=2.43, 95% CI=1.94, 3.04) and Hispanic (OR=1.41, 95% CI=1.07, 1.85) were associated with higher odds of revisiting the ED than White children. In terms of age, children aged 5–11 years (OR=1.78, 95% CI=1.45, 2.20) and 12–14 years (OR=1.17, 95% CI=0.85, 1.82) were associated with higher odds of revisiting the ED than those aged 15–17 years. Medicaid insurance was associated with higher odds of returning to the ED (OR=1.58, 95% CI=1.33, 1.88), whereas being uninsured was associated with lower odds of having ED visits in all 3 years (OR=0.74, 95% CI=0.57, 0.95). Children residing in the lowest-income ZIP code quartile were associated with higher odds of returning to the ED (OR=1.51, 95% CI=1.13, 1.87). In terms of patients’ location, children from small metropolitan areas (OR=0.57, 95% CI=0.38, 0.87), micropolitan areas (OR=0.45, 95% CI=0.26, 0.76), and noncore areas (OR=0.34, 95% CI=0.18, 0.62) were associated with lower odds of revisiting the ED in all 3 years than children residing in large central metropolitan areas.

## DISCUSSION

This study examined the factors associated with returning to an ED for additional visits among pediatric patients with asthma who had an index ED visit in 2016. This cohort of children was followed for 3 subsequent years (2017–2019) to evaluate the persistence of ED revisits. This study extends previous research on ED revisits for asthma that was limited to the analysis of patients within 14 days to 18 months after their index visit.^3^^,^^8^^,^^30^^,^^31^

Three main findings were identified. First, for children with asthma, ED revisits can persist for several years. Almost 32% of children who visited the ED for asthma in 2016 returned for additional ED asthma visits between 2017 and 2019. Although the majority of those who returned revisited the ED in only 1 follow-up year, nearly 11% returned in 2 or more years, and 3% returned in all 3 follow-up years. This long-term return rate is higher than estimates reported in studies examining the rate of return within a few weeks after an index date.^3^^,^^8^ One might assume that a higher rate of return suggests lower quality of ED care during prior visits. However, it has been shown that ED return visits are not necessarily associated with worse clinical outcomes than index visits.^32^ Instead, they may reflect challenges related to asthma control, access barriers, and social and environmental conditions.^33^

Second, the pattern of revisits over the years suggests that higher ED revisit rates occur in the year closest to the index year. This finding is consistent with the natural progression of the disease^31^ and may signify the importance of interventions to promote continuity of care for children discharged from the ED.^34^ Although outpatient follow-up care within 2 weeks of an asthma ED visit could reduce the likelihood of revisiting for asthma in the next year by up to 13%,^8^ it is important to implement solutions that have a longer impact on asthma ED visits.^20^

Third, race/ethnicity, sex, age, type of insurance coverage, income, and residence in an urban area were identified as risk factors for persistent occasional ED revisits for pediatric asthma. Findings from this study align with those from previous studies that showed racial/ethnic and socioeconomic factors as being associated with greater odds of pediatric asthma ED revisits, specifically, factors such as Black race, Hispanic ethnicity, and public insurance.^2^^,^^3^^,^^8^^,^^9^^,^^35^^,^^36^ In particular, the odds of persistently returning to an ED for asthma were high for younger children and those identified as Black or Hispanic and low for those living in fringe metropolitan areas or micropolitan areas. These findings are analogous to those that showed that younger and Black children were more likely to experience emergency revisits within a year of the index admission.^30^^,^^33^^,^^37^^,^^38^ These disparities likely arise from structural inequities related to socioeconomic disadvantage, discriminatory treatment in healthcare settings, and environmental risk. For example, Black and Hispanic children are more likely than White children to reside in neighborhoods with higher exposure to air pollution, including proximity to major roadways and industrial facilities, which can worsen air quality and increase asthma risk.^33^^,^^39^^,^^40^

### Limitations

Although these findings add to the evidence base of health inequities for specified groups of children with asthma and call for preventative interventions, they come with a few limitations. First, the analyses were limited to 3 states from the HCUP-SEDD data that included an individual linkage indicator that allowed the authors to follow a patient across time within a state. Therefore, ED visits that occurred outside the state of residence were not observed. Children who relocated across state lines, sought care in neighboring states, or received care at nonparticipating facilities may therefore have revisited during the study period but were not captured in the analyses. Such incomplete capture of follow-up care would likely attenuate estimated effects.^41^^,^^42^ Second, owing to transitioning into adulthood during the follow-up period, some degree of loss to patient follow-up was possible. Third, variations in state coding or reporting requirements and practices may have impacted findings and led to inconsistencies in longitudinal data analysis. Specifically, how race/ethnicity is recorded in the HCUP-SEDD can bias disparities estimates. Fourth, the short timeframe of the study and the lack of comprehensive patient histories could be a limitation in setting up the reference group, meaning that the patients in the index year of 2016 could have had ED visits in prior years, which were not captured in this study. Therefore, assuming that they only visited the ED in 2016 may introduce a bias in the findings. This limitation may affect the definition of the reference group and could bias estimates toward underestimating the persistence of ED use. Moreover, differences in local health systems, reporting practices, environmental conditions, and access to outpatient care may limit the generalizability of findings to other states or national populations. Finally, estimates may be biased toward poorer families who are more likely to remain geographically stable and thus be captured in the data over time, compared with wealthier families who may relocate and not be observed.

## CONCLUSIONS

This study describes the associations between individual characteristics and asthma‑related ED revisits. Specifically, the findings indicate that Black and Hispanic children, male children, children aged <15 years, those covered by Medicaid, and children residing in low-income ZIP codes and central large metropolitan areas are associated with higher odds of returning to the ED for asthma care, even several years after the index visit, than their respective reference groups.

These findings have some implications for policy and practice. Persistent reliance on the ED for treatment of pediatric asthma implies that health systems should offer interventions and be involved in efforts that reduce the likelihood of children returning to the ED for asthma visits.^43^ Second, by recognizing the risk factors that put children at the highest risk of additional visits, particularly in historically disinvested neighborhoods, providers can extend their services to these groups beyond the provision of acute stabilization alone. Extending services such as timely outpatient follow-up, care navigation, or referrals to community-based resources for high-risk children may help support improved asthma management and potentially reduce reliance on the ED over time.^33^

Further research using large claims databases, such as the national Medicaid sample, is needed to address limitations related to patient follow-up and representation over time and to account for other factors, such as an individual’s medication adherence or environmental exposures and upstream structural inequities.

## CRediT authorship contribution statement

**Nima Khodakarami:** Conceptualization, Methodology, Formal analysis, Software, Writing – original draft, Supervision. **Gogoal Falia:** Conceptualization, Methodology, Writing – original draft, Validation. **Hye Jin Park:** Conceptualization, Writing – review & editing. **Marvellous Akinlotan:** Writing – review & editing. **Alva O. Ferdinand:** Conceptualization, Writing – review & editing, Resources, Validation, Supervision.
